# Three-Dimensional Gradients of Cytokine Signaling between T Cells

**DOI:** 10.1371/journal.pcbi.1004206

**Published:** 2015-04-29

**Authors:** Kevin Thurley, Daniel Gerecht, Elfriede Friedmann, Thomas Höfer

**Affiliations:** 1 Division of Theoretical Systems Biology, German Cancer Research Center, Heidelberg, Germany; 2 Institute for Theoretical Biology, Charité-Universitätsmedizin, Berlin, Germany; 3 Department of Pharmaceutical Chemistry, University of California, San Francisco, San Francisco, California, United States of America; 4 Institute for Applied Mathematics, University of Heidelberg, Heidelberg, Germany; 5 Bioquant Center, University of Heidelberg, Heidelberg, Germany; Emory University, UNITED STATES

## Abstract

Immune responses are regulated by diffusible mediators, the cytokines, which act at sub-nanomolar concentrations. The spatial range of cytokine communication is a crucial, yet poorly understood, functional property. Both containment of cytokine action in narrow junctions between immune cells (immunological synapses) and global signaling throughout entire lymph nodes have been proposed, but the conditions under which they might occur are not clear. Here we analyze spatially three-dimensional reaction-diffusion models for the dynamics of cytokine signaling at two successive scales: in immunological synapses and in dense multicellular environments. For realistic parameter values, we observe local spatial gradients, with the cytokine concentration around secreting cells decaying sharply across only a few cell diameters. Focusing on the well-characterized T-cell cytokine interleukin-2, we show how cytokine secretion and competitive uptake determine this signaling range. Uptake is shaped locally by the geometry of the immunological synapse. However, even for narrow synapses, which favor intrasynaptic cytokine consumption, escape fluxes into the extrasynaptic space are expected to be substantial (≥20% of secretion). Hence paracrine signaling will generally extend beyond the synapse but can be limited to cellular microenvironments through uptake by target cells or strong competitors, such as regulatory T cells. By contrast, long-range cytokine signaling requires a high density of cytokine producers or weak consumption (e.g., by sparsely distributed target cells). Thus in a physiological setting, cytokine gradients between cells, and not bulk-phase concentrations, are crucial for cell-to-cell communication, emphasizing the need for spatially resolved data on cytokine signaling.

## Author Summary

The adaptive immune system fights pathogens through the activation of immune cell clones that specifically recognize a particular pathogen. Tight contacts, so-called immunological synapses, of immune cells with cells that present ‘digested’ pathogen molecules are pivotal for ensuring specificity. The discovery that immune responses are regulated by small diffusible proteins – the cytokines – has been surprising because cytokine diffusion to ‘bystander’ cells might compromise specificity. It has therefore been argued that cytokines are trapped in immunological synapses, whereas other authors have found that cytokines act on a larger scale through entire lymph nodes. Measurements of cytokine concentrations with fine spatial resolution have not been achieved. Here, we study the spatio-temporal dynamics of cytokines through mathematical analysis and three-dimensional numerical simulation and identify key parameters that control signaling range. We predict that even tight immunological synapses leak a substantial portion of the secreted cytokines. Nevertheless, rapid cellular uptake will render cytokine signals short-range and thus incidental activation of bystander cells can be limited. Long-range signals will only occur with multiple secreting cells or/and slow consumption by sparse target cells. Thus our study identifies key determinants of the spatial range of cytokine communication in realistic multicellular geometries.

## Introduction

Cell-to-cell communication is a defining property of multicellular organisms. In particular, the release, sensing and uptake of cytokines, small signaling proteins, by cells is essential for the regulation of the mammalian immune system [1]. Prominent quantitative characteristics of cytokine signaling are high receptor specificity (with *K*_*d*_ ≈ 10^−10^ nM) and low free cytokine concentrations in the picomolar range [2,3]. The physiological cytokine milieu regulates critical processes like the type and strength of the immune response. Quantitative understanding of such cytokine-driven cellular decisions is beginning to emerge [4–8], yet the underlying spatio-temporal cytokine dynamics remain poorly understood. Cytokines act in a heterogeneous environment, typically with high cell-densities. It is not known how they diffuse under such conditions and, in turn, regulate immune responses. Specifically, how far cytokines can signal away from the producing cell is not clear. Perona-Wright et al. [9] have found that interleukin(IL)-4 is seen by most T cells in the lymph node upon parasite infection, including non-specific ‘bystander’ cells. In this case, many T cells throughout the lymph node could be IL-4 producers. By contrast, several observations suggest more localized cytokine communication [4,10–13].

Given the low measured cytokine concentrations, which are often below 10 pM, the question arises whether and how effective paracrine signals are possible at all, in a situation where only a certain fraction (~25%) of the cells secrete cytokine molecules. Of note, 1 pM is about 1 molecule in 1700 μm^3^, compared to ~500 μm^3^ volume of a typical lymphocyte. Higher, systemically elevated cytokine levels arise only in certain immunopathologies, so-called ‘cytokine storms’, where they cause severe damage [14]. However, it has been demonstrated that cytokine concentrations are not always well mixed, and locally higher cytokine concentrations can occur also in ex vivo T cell cultures [12]. Therefore, we asked how and under which conditions such cytokine gradients arise, and if they are able to explain effective paracrine signals.

One possibility to enrich cytokine concentrations would be localized signaling to specific target cells by an immunological synapse [15–18]. Immunological synapses are formed between immune cells by surface proteins after antigen recognition [15,16,19]. They have been observed between various cell types of the immune system, including immunological synapses between T cells and antigen presenting cells (APC, e.g. B cells [10] and dendritic cells [20]), and immunological synapses between T cells and T cells [12]. Many cytokines are secreted preferentially into the immunological synapse [10,21–23], and a range of high-affinity cytokine receptors have been found to be specifically located in the immunological synapse, too [11]. Therefore, it is likely that the synapse has an important function for cytokine signaling, beyond its role for T cell receptor signaling on which theoretical studies have focused [24,25]. Cytokine signaling through immunological synapses might also explain the pleiotropic effects observed for most cytokines, as it would provide specificity of cytokine signaling by restriction of their action. Nevertheless, it is unlikely that paracrine cytokine signals are only possible between cells that are directly connected by an immunological synapse. For instance, Sanderson et al. [26] found that interferon-γ can be seen by bystander cells other than the target cells to which the synapses are formed. To understand which parameters govern autocrine versus paracrine cytokine signaling, we analyzed in this study reaction-diffusion models of cytokine signaling at two scales: through the immunological synapse between two cells and in three-dimensional arrays of many (>100) cells.

For this purpose, we chose the cytokine interleukin(IL)-2 as a model system, a cytokine showing polarized secretion and corresponding receptor expression [10,11,22]. IL-2 was first identified as a T cell growth factor [27], but, paradoxically, is a critical mediator of immune tolerance [28–31]. It is secreted by T helper (Th) cells early after antigenic stimulation and taken up by high-affinity IL-2 receptor (IL-2R) on Th cells and regulatory T (Treg) cells [28–31]. Treg cells mediate immune tolerance and are critical for the prevention of autoimmune reactions [32,33]. IL-2 secretion is digital, i.e. upon receiving an antigen stimulus, only about one quarter of a Th cell population releases IL-2 molecules [34–36]. It is an open question if IL-2 and other cytokine signals act in an autocrine or paracrine manner [31,37]. In response to IL-2 uptake, Th cells and Treg cells upregulate CD25, the α-subunit of the IL-2R. CD25 is often used as an activation marker of T cells, because it precedes proliferation of Th cells and subsequent recruitment of effector immune cells [30,38]. Although IL-2 secreting Th cells upregulate CD25, Long and Adler [37] reported that they lack phosphorylated STAT5, a key intermediate in the IL-2R signal transduction cascade. In the same experiment, other Th cells not secreting IL-2 also upregulate CD25 in response to IL-2, and in addition show fully functional signal transduction [5,37]. These data suggest that the dominant mode of IL-2 signaling is paracrine, in contrast to the presumed function of the immunological synapse in containing secreted cytokines [16,17]. However, unlike T cells, most APC do not express functional IL-2 receptor (IL-2R) [39]. Thus, both the study by Sanderson et al. [26] and the properties of IL-2 signaling suggest a role of the immunological synapse for cytokine signals that goes beyond signal amplification between the two cells associated by a synapse.

In this study, we addressed the question of how and under which conditions paracrine cytokine signals occur despite the measured low bulk concentrations in the picomolar range, and we aimed to define the parameters that control the range of cytokine signaling. To this end, we considered the two key spatial scales, the sub-μm scale of the immunological synapse and the supra-μm scale of cell-to-cell communication. We investigated reaction-diffusion models on these two scales by analytical techniques and advanced finite-element computations in three spatial dimensions [40–44]. To be specific, we utilized a simple, experiment-based mathematical model for IL-2 signaling and gained more general insight through systematic variation of parameters. Our results show that paracrine cytokine signaling is possible in the presence of local concentration gradients combined with nonlinear signal amplification. The spatial range of cytokine signaling can be tuned from purely autocrine via intrasynaptic and short-range paracrine to long-range paracrine. For a wide array of parameters, we found that cytokine gradients in dense multicellular environments range over one to few cell diameters. These computational findings can inform novel experiments probing the spatio-temporal dynamics of cytokine signaling [45].

## Results

### Target cell density and receptor expression control paracrine cytokine signals

**Fig 1:**
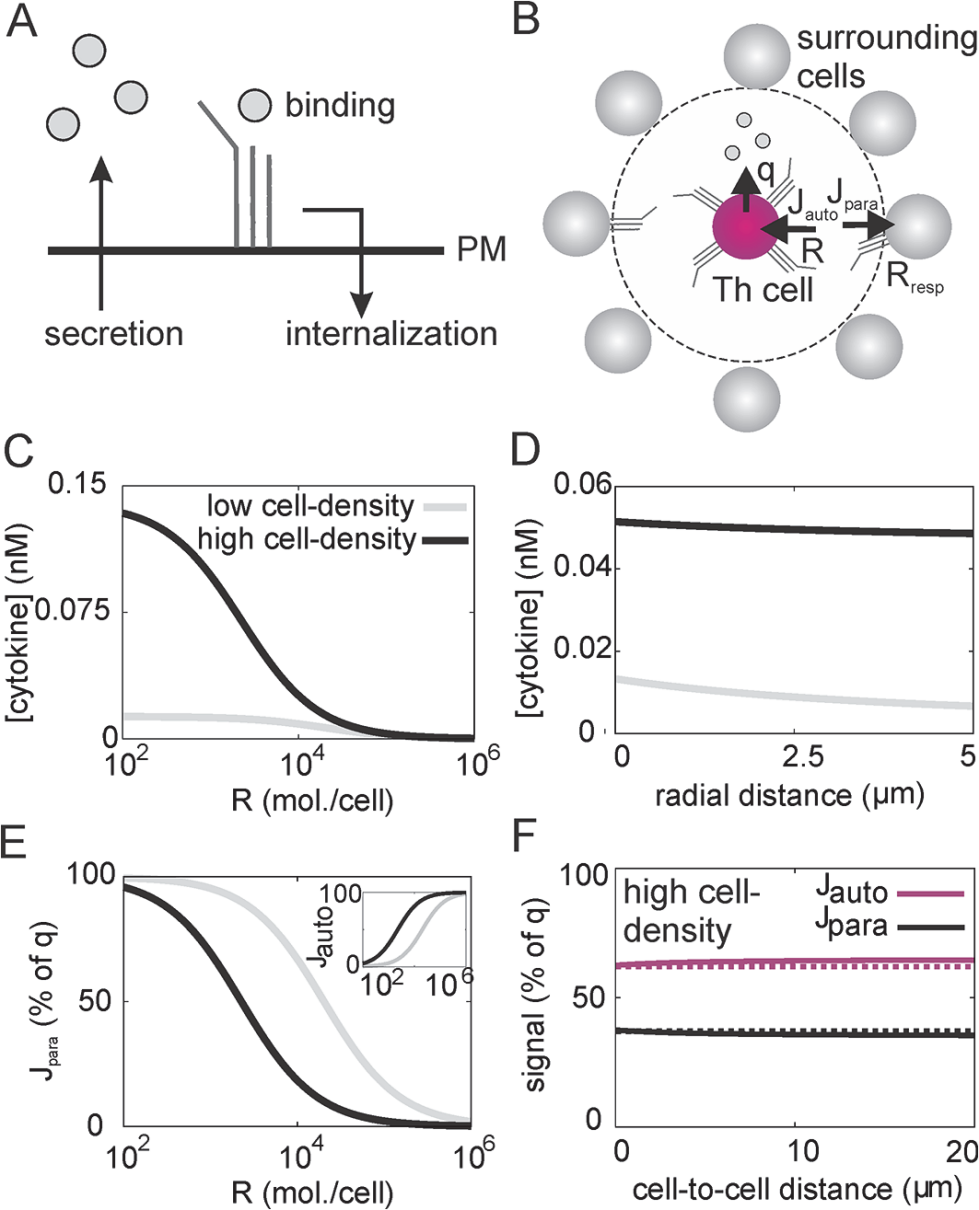
Paracrine cytokine signals depend on cell density and receptor number. (A) Cytokine secretion and uptake is followed by receptor internalization, leading to a reduced cytokine concentration in the medium. (B) Schematic of the high cell-density scenario. A cytokine secreting cell is surrounded by a layer of responder cells that provide a diffusion barrier for the cytokine. Signaling can be autocrine (J_auto_), i.e. cytokine molecules are bound by receptors of the cytokine secreting cell, or paracrine (J_para_), i.e. bound by receptors on responder cells. (C) Cytokine concentration profile in the model with homogeneous secretion and uptake. (D) Cytokine concentration 1 μm away from the cytokine secreting cell, in the limits of high and low cell-density. (E) Paracrine signal J_para_ and autocrine signal J_auto_ (inset) as fraction of secreted cytokine molecules. (F) Autocrine and paracrine uptake in the high cell-density scenario. Dotted lines are approximations in the limit of fast diffusion (see S1 Text). Parameter values: R = 4000 mol./cell, and see Table 1.

The binding of cytokines to their high-affinity receptors is followed by receptor internalization and intracellular cytokine degradation, so that cytokine molecules are removed from the medium (Fig 1A). Thus, regulating the strength of cytokine signaling by cytokine receptor expression might also affect the extracellular cytokine concentration and hence, indirectly, signaling. To gain quantitative insight, we first studied a simple reaction-diffusion model, where a cytokine-secreting cell is surrounded by cells that can take up the cytokine. To allow for an analytical solution, we assume the surrounding cells to be placed on a spherical shell with the secreting cell in the center (Fig 1B, see Materials and Methods). For convenience, parameter values are summarized in Table 1. If the target cells are located far away (i.e., their density is low), the cytokine concentration experienced by the target cells is nearly independent of the level of receptor expression (Fig 1C) because the dilution of the cytokine occurs primarily by diffusion in the three-dimensional tissue. On the other and, if the density of target cells is so high that they immediately surround the cytokine secreting cell, the cytokine concentration is practically homogeneous in the small intervening space, as the timescale of diffusion over such a short distance is fast compared to the timescale of cytokine uptake (Fig 1D and S1 Text). As a consequence, the cytokine concentration experienced by proximal target cells is set by the balance of secretion rate by the cytokine-producing cell and uptake rate. The autocrine and paracrine uptake rates *J*_auto_ and *J*_para_ depend on the level of cytokine receptor expression on the target cells (Fig 1E), and are practically independent of the cell-to-cell distance even at high cell density (Fig 1F; the low cell-density scenario is independent of the cell-to-cell distance by construction). Interestingly, cytokine concentration (Fig 1C) and uptake rates (Fig 1D) are sensitive to receptor expression on proximal targets cells in the physiologic range of 100 to several 1000 receptor molecules per cell [5]. Thus, this simple model indicates that with a high density of target cells, cytokine receptor expression controls the amount of paracrine cytokine signal.

**Table 1:** Symbols and parameter values.

Parameter	Symbol	Value	Unit	Reference
IL-2 diffusion constant	D	10	μm^2^/sec	[4]
T cell radius	ρ	5	μm	
Cell-to-cell distance (surface to surface)	L	5	μm	
IL-2 secretion rate	q	10	Molecules/sec	[35]
IL-2/IL-2R binding rate	k_on_	111.6	1/(nM h)	[4]
IL-2 degradation rate	k_d_	0.1	1/h	[4]
Synapse contact area radius	a	2	μm	[11]
Synaptic distance	L	20	nm	[34]
Basal IL-2R number	IL-2R^low^	100	Molecules/cell	[4,5]
Upregulated IL-2R number	IL-2R^high^	4000	Molecules/cell	[4,5]
Fraction of IL-2 secreting cells		25	%	[35]
Basal IL-2R expression rate (Th cells)	v_0_ (Th)	150	Molecules/cell/h	[4]
Basal IL-2R expression rate (Treg cells)	v_0_ (Treg)	1000	Molecules/cell/h	[4]
IL-2 induced IL-2R expression rate (Th cells)	v_1_ (Th)	3000	Molecules/cell/h	[4]
IL-2 induced IL-2R expression rate (Treg cells)	v_1_ (Treg)	8000	Molecules/cell/h	[4]
Half-saturation constant of IL-2R expression	K	1000	Molecules/cell	[4]

### The immunological synapse controls type and strength of cytokine signals

**Fig 2:**
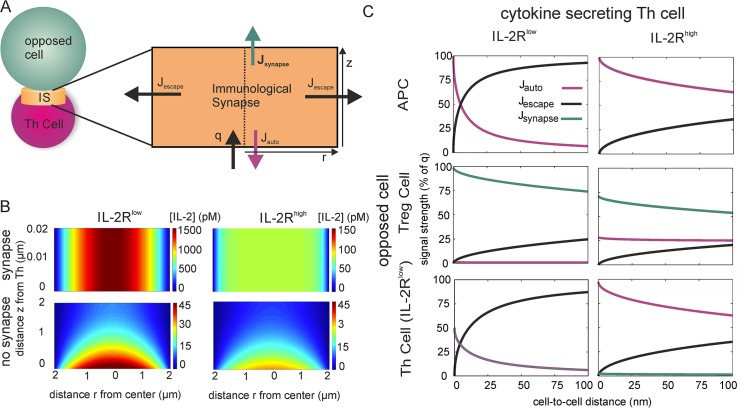
Polarized cytokine secretion and cytokine receptor expression can suppress paracrine signals. (A) Model: Cytokine molecules are released into a cylindrical region (radius of contact area: 2μm) between Th cell and an APC or other opposed cell. Molecules can be taken up by cytokine receptors on the cytokine secreting cell (autocrine signal J_auto_) or on the opposed cell (synaptic signal J_synapse_), and molecules reaching the outer boundary escape (signal J_escape_, ‘effective secretion rate’) and do not return. (B) Cytokine concentration in the immunological synapse (cylinder depicted in A). IL-2R^low^: R = 100; IL-2R^high^: R = 4000; coordinates z and r refer to the position inside the cylinder as defined in panel A. ‘Synapse’: Tight synapse with synaptic distance 20nm. ‘No synapse’: Cell-to-cell distance of 2μm, i.e. no synapse is formed. (C) Distribution of cytokine signals in the cases of IL-2R^low^ or IL-2R^high^ cytokine secreting cells, for different types of opposed cells: APCs do not express cytokine receptors (R_resp_ = 0), while Treg cells express high levels (R_resp_ = 10^4^ mol./cell) and opposed Th cells express basal levels (R_resp_ = 100 mol./cell) of IL-2R. Note that J_synapse_ does not exist if the opposed cell is an APC, and J_synapse_ equals J_auto_ in the case of a T-T synapse with two of IL-2R^low^ Th cells (bottom left).

The model of the previous section assumed homogeneous secretion of the cytokine over the cell surface. However, T cells release IL-2 and other cytokines in a polarized fashion into the immunological synapse [10,21–23]. Therefore, we analyzed a model of cytokine secretion and uptake in the immunological synapse, represented by a small cylindrical region between a Th cell and an opposed APC or second T cell (Fig 2A, see Materials and Methods), extending previous work [46]. The distance between Th cell and opposed cell, in the following referred to as synaptic distance, is in the range of 10 to 40 nm [19,47]. This close contact between Th cell and opposed cell causes a cytokine concentration profile which is almost homogeneous between the two cells in the center of the synapse, and sharply falls off towards the outer boundaries through which cytokine molecules are lost practically irreversibly (Fig 2B, top). In the case of low receptor expression (Fig 2B, top left), the cytokine concentration reaches values in the nM range. Thus, the synaptic cytokine secretion results in locally much higher concentrations than homogeneous secretion (see Fig 1C), in line with experimental data [12]. For comparison, consider cytokine secretion into a cylindrical region with length 2 μm, a typical value for nearby cells but much larger than the immunological synapse (Fig 2B, bottom). In this case, the cytokine concentration falls to less than 20 pM at the surface of the opposed cell. Hence, the very small synaptic distance in a fully formed immunological synapse is crucial for the establishment of high local cytokine concentrations.

The immunological synapse causes two conceptually different types of paracrine signals: Cytokine molecules may bind to cytokine receptors at the opposed cell (*J*_synapse_) or escape into the extracellular space (*J*_escape_), potentially reaching other nearby cells. Cytokine molecules may also induce autocrine signals by binding to receptors at the secretory cell (*J*_auto_). Fig 2C shows the fractions of *J*_auto_, *J*_synapse_ and *J*_escape_, choosing IL-2 receptor densities that are characteristic of naïve (IL-2R^low^) or preactivated (IL-2R^high^) IL-2 secreting T cells, and for opposed cells with different IL-2R expression. IL-2R^high^ cells recapture most of the secreted IL-2 molecules, irrespective of the type of opposed cell and the synaptic distance.

Naïve, IL-2R^low^ cells show a strong dependence on the synaptic distance (Fig 2C, left). If the synaptic space is sufficiently narrow, *J*_escape_ is small; the escape flux could be even further reduced by adhesion molecules sealing off much of the synapse from the extracellular space. If the opposed cell is a second T cell, then the secretory cell and the opposed cell compete for the cytokine molecules. Treg cells outcompete Th cells due to their large receptor number. On the other hand, APCs do not express IL-2R and the IL-2 signals would be purely autocrine. For synapses with somewhat larger synaptic distances, a considerable amount of cytokine molecules can escape (enhanced *J*_escape_) and provide paracrine signals to surrounding cells. Interestingly, the ratio of *J*_auto_ and *J*_escape_ is most sensitive to the synaptic distance in the physiologic range between 10 nm in the close-contact zone and 40 nm in the outer region of the immunological synapse [19,47]. In all cases, the fraction *J*_escape_ of cytokine molecules that escape for paracrine signaling is considerably smaller than in the case of homogeneously distributed cytokine secretion and receptor expression (Figs 2C and 1D). However, even if the cytokine secreting cell is pre-activated and hence autocrine IL-2 uptake is high (IL-2R^high^), a sizeable fraction of cytokine molecules still diffuses out of the synapse (*J*_escape_ ~20%).

In summary, our model reveals two main implications of an immunological synapse for cytokine signaling: A tight synapse causes highly localized cytokine distributions, and it enhances the probability of autocrine recapture. Both properties result from the high aspect ratio of the radius of the cell contact area and the synaptic distance (r and z in Fig 2A). The two properties have opposing effects on the strength of paracrine cytokine signals: While localized cytokine concentrations increase the likelihood of a local paracrine signal, the reduction in effective cytokine secretion reduces the potential of paracrine signals.

### In-silico Th cell culture exhibits localized paracrine IL-2 signaling

The analytically tractable models gave insight into the qualitative properties of paracrine cytokine signaling, and they made quantitative predictions on the consequences of the various time and length scales in the system. For example, the high aspect ratio of the immunological synapse evokes highly localized cytokine concentrations in the vicinity of cytokine secreting cells resembling secretion from a point source (see Fig 2B), and the high diffusion constant in relation to the receptor dynamics makes the system largely independent of the cell-to-cell distance (Fig 1E and 1F). However, the simple models studied above cannot answer the question if effective paracrine signals are possible despite the low bulk cytokine concentrations. To illustrate this problem, consider a classical formula from Berg and Purcell for the timescale of ligand diffusion towards a receptor [48] (Materials and Methods). Measured cytokine concentrations in serum or in supernatants of *ex-vivo* T cell cultures are typically in the picomolar range [2,3]. Assuming a spatially uniform cytokine concentration of 10 pM and a receptor number of 100 per cell, as is typical for the high-affinity IL-2R on naïve T cells, that calculation reveals that on average, every 7 min a receptor becomes bound by a cytokine molecule. Under these conditions it would take hours to induce a reliable signal, indicating that bulk cytokine concentrations might just be capable of, or even be too low for, stimulating signal transduction. However, it has been reported that IL-2 is subject to appreciable spatial gradients, with much higher concentrations at the surfaces of T cells [12,49].

**Fig 3:**
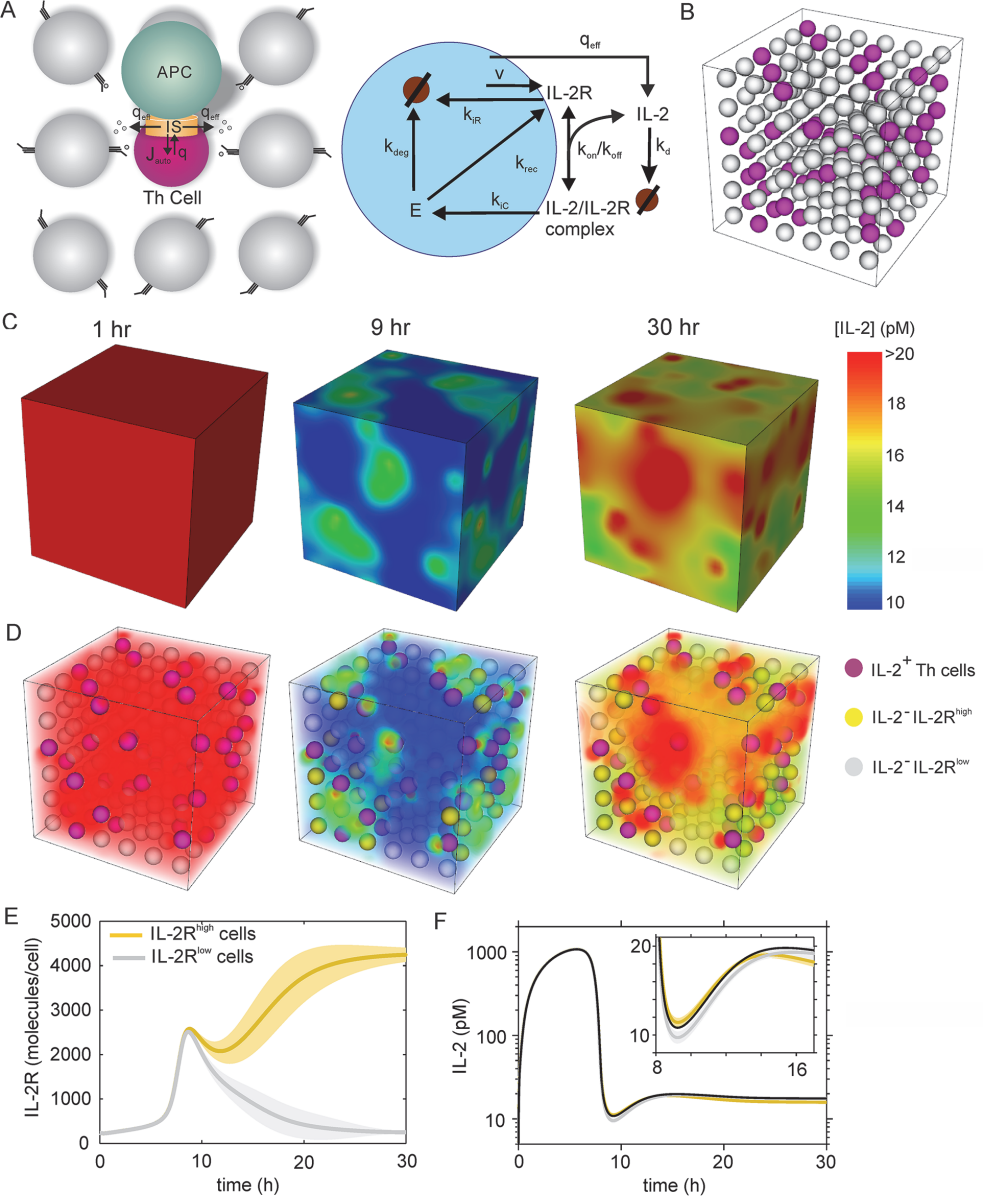
Spatiotemporal cytokine dynamics of an in silico Th cell population. (A) Model scheme: (left panel) A fraction of Th cells (about 25%) releases cytokine molecules into the immunological synapse with rate q. Most of these molecules bind to the polarized cytokine receptors on the cytokine secreting cell (see Fig 2), a smaller fraction (about 20%) escapes the synapse and is considered as effective, polarized secretion rate q_eff_. (right panel) Intracellular receptor dynamics include receptor expression (rate v), binding of cytokine molecules (k_on_, k_off_), internalization of free and bound cytokine receptor (k_iR_, k_iC_), receptor recycling (k_rec_) and receptor degradation (k_deg_). Further, IL-2 degradation with rate k_d_ is considered. (B) Setup of the simulation of a T cell population in three dimensions. The cell-to-cell distance (shortest distance between cell surfaces) and cell radius are both 5μm. The simulations included 216 cells, of which 54 were randomly chosen as secretory (marked pink). Only T cells are included in the simulation, APCs are assumed to fill the region between T cells, and by that induce synapse formation leading to polarized effective secretion at randomly chosen surface points. Only non-secretory T cells express IL-2R in the simulations, because receptors of IL-2 secreting cells are considered in terms of the reduced, effective secretion rate. (C-D) IL-2 concentration at indicated time points after starting the simulation. 59 of the 162 non-secretory cells (see B) were activated, defined by expression of more than 4000 receptors after 30h simulation time (marked gold). (C) No transparency, only the surface of the cubic region is visible. (D) Limited transparency allows viewing concentration profiles inside the region. (E-F) Time course of the receptor number (E) and average IL-2 concentration at the surface (F) of activated and not activated Th cells from the simulation in C-D. Solid lines indicate averages, blurred region standard deviations, and the black line in (F) is the average IL-2 concentration in the simulated region.

To investigate the origins and consequences of spatially inhomogeneous dynamics of cytokine signaling, we performed extensive three-dimensional simulations of a T cell population (Fig 3A and 3B). As before, we focus on the cytokine IL-2, for which many parameters, including secretion and receptor expression rates, have been estimated from experiments [5,35,50], and experimentally tested models for the IL-2R dynamics are available [4,5,7]. To account for polarized secretion at the immunological synapse, we do not explicitly model synapse formation but consider the effect of discrete IL-2 sources from which IL-2 escapes into the extra-synaptic space (with rate *q*_eff_, corresponding to *J*_escape_ in the simplified model of the previous section). The position of the IL-2 source of a producing cell is a randomly chosen point at the cell surface. IL-2 secretion is all-or-nothing [34,36]: only about one quarter of antigen-stimulated T cells release IL-2 molecules, and among these cells, the IL-2 secretion rate is in the range of 10 molecules per second [35]. In accordance with experimental data, already activated IL-2 secreting cells have high IL-2R expression which, for simplicity, we take as constant [37]. Non-secreting cells are assumed to upregulate IL-2R expression in response to IL-2 homogeneously at their cell surface.

Consistent with experimental data [39], APC themselves do not express IL-2R but constitute simply ‘excluded volumes’ with respect to the IL-2 dynamics. To focus on the role of IL-2 uptake by T cells, we do not consider the APC explicitly, but only its consequences for polarized secretion and uptake (see above) (Table 1). Despite this simplification, our simulations consider realistic extracellular volumes (as determined by the cell distances) between the cells as a basis for determining the extracellular concentrations of the secreted cytokines.

Based on these assumptions, we simulated the IL-2 dynamics for a large number of T cells (216 cells in a volume of ~1 nl, Fig 3). Stimulating IL-2 secretion in a fraction of T cells (Fig 3A and 3B), the IL-2 concentration increases rapidly and nearly homogeneously for several hours after stimulation (Fig 3C and 3D and S1 Fig). Then, in response to the high IL-2 concentration resulting from paracrine signaling, IL-2R expression is upregulated in non-secreting cells (Fig 3E) and causes fast IL-2 uptake from the medium. As a result, concentration gradients occur: In large parts of the simulated region, the IL-2 concentration reaches a steady-state at around 10 pM while locally it is more than twice as large (Fig 3C, red regions at 30 h). This inhomogeneity in IL-2 concentration corresponds to receptor upregulation (activation) of non-secreting Th cells: IL-2R^high^ cells are found near the regions with high IL-2 concentration. Analysis of the time course (Fig 3E) shows that all cells upregulate IL-2R levels in response to the increased IL-2 concentration in the first hours after antigenic stimulation and IL-2 secretion. However, as the high-affinity IL-2R is being upregulated, IL-2 becomes increasingly depleted in the medium. As a result, only a fraction of the cells receive a sufficient IL-2 stimulus to sustain high IL-2R expression (IL-2R^high^ cells in Fig 3E), whereas the remaining cells downregulate IL-2R expression (IL-2R^low^ cells).

Interestingly, the time courses of IL-2 concentrations at the surfaces of the cells show only small differences between IL-2R^high^ and IL-2R^low^ cells (Fig 3F): In the beginning, IL-2 equally rises near IL-2R^high^ and IL-2R^low^ cells (see Fig 3D), but as IL-2 depletion sets in, the cells that eventually become IL-2R^low^ cells receive slightly less IL-2. Later, at steady-state, the IL-2 concentration is somewhat higher in the microenvironment of IL-2R^low^ cells, because they do not consume as many IL-2 molecules. This form of local bistability, which occurs in the expression of IL-2R on Th cells, was observed already in Ref. [4]: Based on a quasi-stationary state assumption, Busse et al. showed that in the model without Treg cells, the IL-2R expression rate responds to the increase of the secretion rate in a digital way and the cells are activated only after a certain threshold is exceeded. A small bistable region around the threshold is observed. These findings were supported by experimental data from primary T cells cultured *ex vivo* [4]. Thus our present model with the immunological synapse and 3D diffusion matches the bistable system behavior seen in the simpler analytical model.

Taken together, our simulations indicate that the amount of IL-2 escaping from the immunological synapse is sufficient to sustain paracrine signaling in at least a fraction of surrounding cells. However, competition for the cytokine can cause heterogeneity in the response of a cell population and result in bulk IL-2 levels that are much lower than local concentration peaks and in agreement with concentration levels measured by ELISA (see Discussion).

### Competitive IL-2 uptake by regulatory T cells

**Fig 4:**
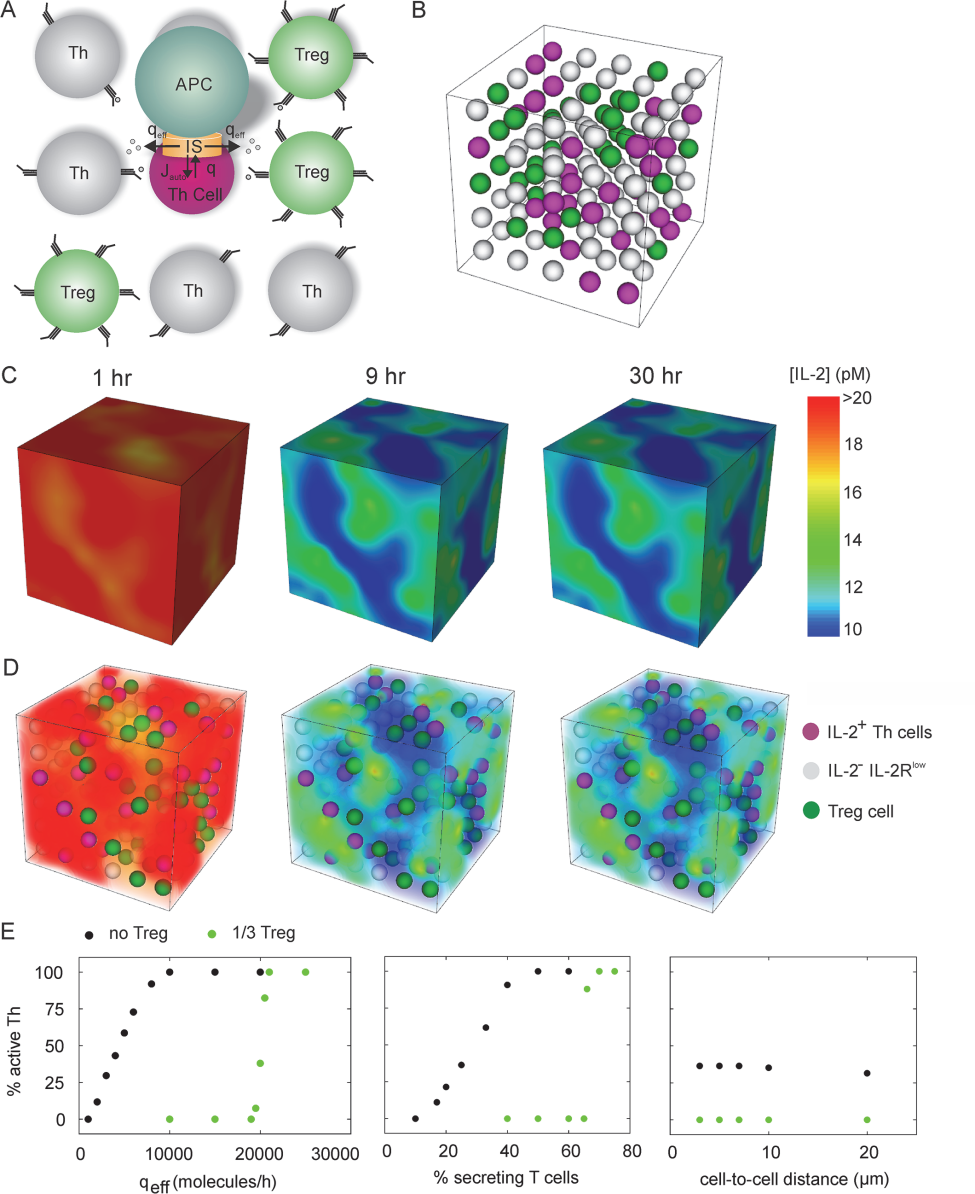
Spatiotemporal cytokine dynamics of an in silico Th-Treg coculture. (A) Model scheme: As in Fig 3A, with addition of constitutively IL-2R^high^ Treg cells. As compared to Th cells, Treg cells have both initially higher receptor number (basal IL-2Rα expression rate) and higher IL-2 induced receptor expression. (B) Setup of the simulation, see Fig 3C. Here, from a total of 216 cells, again 54 are randomly chosen as secretory, and additionally, 54 are randomly chosen Treg cells. (C-D) IL-2 concentration at indicated time points, see Fig 3D and 3E. None of the 108 responder Th cells were activated. (E) Fraction of activated (IL-2R^high^) Th cells after 30h simulation time in the in silico Th cell culture or Th-Treg coculture (see Figs 3 and 4) under various conditions. The paracrine signal increases sharply with the effective secretion rate or the fraction of cytokine secreting cells, but is almost independent of the cell-to-cell distance (shortest distance between cell surfaces).

Regulatory T cells constitutively express high levels of high-affinity IL-2R but do not secrete IL-2 [29,30]. To study the effect of Treg cells on the IL-2 dynamics after activation of conventional Th cells, we simulated a T-cell population consisting of antigen-stimulated IL-2 secreting and non-secreting Th cells as well as Treg cells (Fig 4A and 4B). Compared to the situation in the absence of Treg cells (cf. Fig 3), the IL-2 concentration attains a spatially inhomogeneous steady state more rapidly, with the overall IL-2 concentration being lower (Fig 4C and 4D and S2A Fig). Importantly, the non-secreting Th cells do not permanently upregulate IL-2R in the presence of Treg cells because the Treg cells suppress the paracrine IL-2 signal. The comparison with the simulations without Treg cells (Fig 3) imply that Th cells require for sustained IL-2 signaling both a transient strong and a stable weak IL-2 stimulus. The finding that Th cells can sustain IL-2 signaling at low cytokine concentration, but only after initial stimulation with high cytokine concentration, is a spatio-temporal phenomenon similar to hysteresis: Active cells express more cytokine receptors, which bind more cytokine molecules even at lower concentration and thus stabilize the active state once it is achieved. Treg cells can suppress prolonged IL-2 signaling in Th cells by inhibiting the strong initial IL-2 signal and the resulting upregulation of the high-affinity IL-2R.

Having established that an effective paracrine IL-2 signal is possible in our model, and that it can be suppressed by Treg cells, we analyzed to which extent key parameters shape the spatio-temporal dynamics: IL-2 secretion rate, cell-to-cell distance, and fraction of IL-2 secreting cells. Without Treg cells, the number of activated Th cells increases linearly with the effective IL-2 secretion rate *q*_eff_ until, eventually, all cells in the simulated region become active (Fig 4E, left panel). By contrast, the presence of Treg cells creates a threshold at an effective secretion rate of *q*_eff_ ~ 20000 molecules/h (about 5 molecules/s), below which there is no paracrine IL-2 signaling between Th cells. The same pattern is observed if we vary the fraction of cytokine secreting cells instead of the effective secretion rate (Fig 4E, middle panel), which reflects digital IL-2 secretion [34,36]. Hence the presence of Treg cells changes the paracrine IL-2 dynamics from a gradual to an all-or-none response: Either the paracrine signal is completely suppressed by competitive uptake, or suppression is overrun and all cells are activated. Note that *q*_eff_ measures only the IL-2 molecules that escape from the immunological synapse; assuming a tight synapse, this would only be 20% of the total secretion (see Fig 2). However, measured IL-2 secretion rates are ~10 molecules/s [35,50], which is likely to be too small to titrate out the Treg cells in a physiological setting where IL-2 is secreted into the synapse.

Within the range from 2 to 20 μm [12], the cell-to-cell distance (measured between cell surfaces of neighbored cells) does not influence the amount of Th cells that become activated by the paracrine IL-2 stimulus (Fig 4E, right panel). This is because, as anticipated by the analytically treatable model (see Fig 1F), cytokine molecules can reach nearby cells rapidly by diffusion compared to the slower time scales of changes in IL-2R expression and IL-2 internalization. Thus, the exact cell-to-cell distance is unimportant in the physiological range.

### IL-2 producers surrounded by IL-2-responsive cells produce short-range paracrine signals

**Fig 5:**
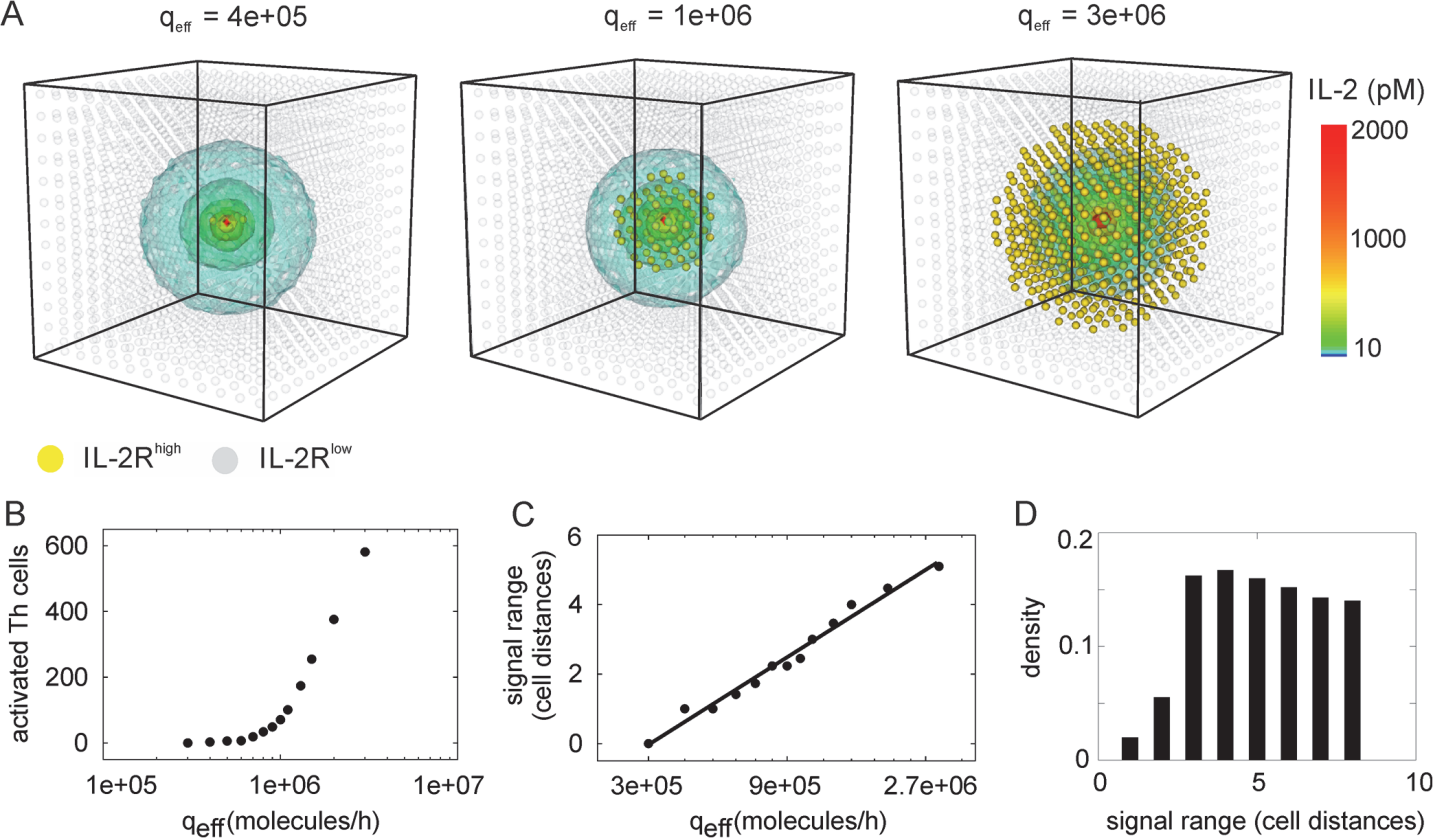
Spatial range of paracrine IL-2 signals. (A) Large-scale simulation (2198 Th cells) with one IL-2 secreting Th cell placed in the center, q_eff_ = 10^6^ molecules/h. 71 Th cells are activated (IL-2R^high^, marked gold). (B) Activated Th cells in simulations as shown in A, for varying values of q_eff_. (C) Spatial range of the effective paracrine signal. Dots are replotted from panel B, with the signal range determined given in cell distances, i.e. by the distance from the center of the region to the most distant activated cell, normalized by the distance between the centers of two cells (15μm). The solid line is a best fit to the function *f*(*x*) = *a* ln(*x*/*x*_0_) (D) Distribution of traveling distances of cytokine molecules. The traveling distance is the distance from the secreting cell at which ligands are bound by receptors. These distances are obtained from a pulsed, homogeneous secretion simulation in a domain of 4913 responding cells (17 in each direction in 3D; see Methods). The traveling distance is shown as signal range is, calculated as in (C).

Our simulations yielded global elevations in IL-2 concentration only transiently before the target cells expressed high levels of IL-2R; beyond this point, only short-range IL-2 gradients were observed, with local concentrations governing IL-2 signaling. Generally, we expect that the balance between cytokine secretion, dilution through diffusion in the three-dimensional extracellular space and cellular consumption will determine the signaling range. To understand the interplay of these three factors, we performed large-scale simulations of an area containing ~2000 cells, with a single IL-2-secreting Th cell surrounded by non-secreting Th cells which all are potential responders to the IL-2 (Fig 5A). Although we use the specific parameters for IL-2 here, this model is of more general interest and applies to other situations with few signaling cells and many responder cells (e.g., IL-4 secreting Th cells in a B cell population [9]), or can be thought of as representing a cluster of several cytokine secreting cells in a population with a small density of cytokine secreting cells elsewhere.

We found that for the secretion rates estimated for IL-2 [35,50], high IL-2 concentrations are restricted to the microenvironment of the cytokine secreting cell (Fig 5B). Remarkably, although secretion is assumed to be polarized through the synapse, the cytokine concentration is higher along the entire surface of the secreting cell, including the pole opposite to the synapse, than at nearby cells. This is due to the absence of IL-2R on the surface of secreting cells (except for the synaptic space). For larger secretion rates (of the order to 10^6^ molecules/h or 280 molecules/s), the IL-2 signal reaches hundreds of cells. However, with the experimental estimate for the IL-2 secretion rate (10 molecules/s [35,50]), of the order of a 100 secreting cells would be needed to realize such a high rate (assuming an effective secretion rate of 10-20% of the total rate, see Figure 2). Therefore, IL-2 from an individual producer will act locally whereas only large clusters of activated cells could cause long-range signals. The occurrence of two distinct spatial signaling regimes as a function of secretion rate is expected because the cellular uptake rate can be saturated by high cytokine concentrations (akin to an enzymatic Michaelis-Menten rate law where the cytokine receptors function as the enzyme). Below saturation the cytokine signal remains local. Interestingly, the spatial range scales linearly with the logarithm of the secretion rate (Fig 5C). Hence the signaling range exhibits a fold-change response to the effective secretion rate (see Discussion).

To further analyze the properties of cytokine diffusion, we computed the traveling distance of cytokine molecules, i.e. the distance from the cytokine secreting cell at which a ligand is taken up by a receptor. For this purpose, we simulated a pulsed, homogeneous stimulation (see Methods) in a region covering ~5000 cells. We found that despite the apparent short-range induction of effective paracrine signaling, the traveling distance has a broad distribution peaking around four cells away from the cytokine secreting cell in each direction (Fig 5D). Thus, in our reaction-diffusion system, the chemical reactions on the cell surface dominate the diffusion and determine the IL-2 gradient formation. We further compared the distribution of traveling distances with earlier analytical expressions obtained from a reaction-diffusion model of morphogen gradient formation [51]. Despite some differences in the model architecture (see Methods), and despite numerical limitations in simulating an ‘infinite domain’ as assumed by the analytical methods of Ref. [51], our simulations are in good agreement with those analytical results (S3 Fig).

Taken together, our simulations of long-range cytokine diffusion and uptake show that long-range paracrine signals are possible in principle, but require exceptional circumstances (extremely high rates of cytokine production or large clusters of cytokine producing cells) that might not readily occur *in vivo*.

## Discussion

The spatial regulation of cytokine signaling in the immune system has spurred much interest, particularly in relation to the specificity of cytokine action [4,9–12,22,23,26]. Experimentally, however, cytokine signaling has not been probed directly at fine spatial resolution, although recent advances in synthetic biology could provide new tools in the near future [45]. Here, we used a computational approach to study cytokine signaling in realistic three-dimensional geometries. To this end, we considered two distinct spatial scales. First, we analyzed polarized signaling across narrow junctions – immunological synapses – between immune cells (nm scale). We find that synapses enhance autocrine signaling and signaling towards the cell connected by the synapse, but, importantly, cannot prevent substantial cytokine escape for paracrine communication by mere geometry. Second, we employed advanced simulation tools for partial differential equations to dissect the dynamics of this ‘spill-over’ paracrine signal in dense ensembles of hundreds of communicating cells (μm scale). Using experimentally established parameters for the T-cell cytokine IL-2, we find that cytokine signals emanating from producing cells are short-range (one to few cell-to-cell distances) because of uptake by target cells or competitors. Long-range communication requires coherent secretion by tens to hundreds of producers or/and sparse uptake. Thus we predict that gradients at the cellular length scale are a key property of cell-to-cell communication by cytokines.

We note that the spatial range of diffusible signals is also of relevance for morphogen action [52,53]. In contrast to immune cell signaling with a typical time scale of many hours during which diffusive gradients reach steady state, the transient behavior on shorter time scales is of particular interest for morphogen gradients [51,54].

Cytokine concentrations as measured by ELISA studies in cell supernatants are typically very low, in the picomolar range [3,5,49]. As low cytokine concentrations would imply long signaling times (see Eq 1 below), we hypothesized that paracrine cytokine signals rely on much higher cytokine concentrations in the microenvironment of target cells, which have indeed been detected by live cell imaging [12]. However, it is generally believed that cytokine signaling occurs in the regime of fast diffusion, which is reflected by our parameter values—the typical spatial range of fast diffusion, $\sqrt{D/k_{d}}$ (see e.g. [55]), spans 40 cells away from the cytokine secreting cell for our values (see Table 1). Therefore, we analyzed the spatiotemporal dynamics of cytokine signals by more detailed mathematical modeling and simulations. We found that spatial gradients do occur due to nonlinear receptor dynamics and polarized IL-2 secretion at the immunological synapse, despite fast diffusion. This was also quantified, e.g. in terms of the traveling distance of cytokine molecules (Fig 5D). We showed by extensive simulations in three spatial dimensions that such cytokine gradients can mediate paracrine signals targeting cells other than those connected by the immunological synapse, as previously suggested for interferon-γ [26]. Moreover, we analyzed the parameters that control paracrine signaling on the different spatial scales.

It is a long-standing question if cytokine signals are predominantly autocrine or paracrine. IL-2 has initially been thought of as a prototypical autocrine signal facilitating self-activation of Th cells [27,30,56]. More recently, paracrine IL-2 signaling towards Treg cells was identified as essential to prevent autoimmune diseases [29,31], possibly due to competition with autocrine self-activation [4,5,28,57]. Recent experimental observations suggest that also paracrine IL-2 signals towards other Th cells are important for regulation of immune responses, while true autocrine IL-2 signals are suppressed by the intracellular signal transduction pathway [5,37]. A plausible explanation would be that IL-2 secreting cells are constitutively activated, i.e. prone to proliferation and differentiation, due to a strong signal from the T cell receptor, and do not rely on signals via the IL-2R. Th cells not secreting IL-2 may have received a weaker T cell receptor signal, and are only fully activated if they receive additional stimulation from the IL-2R. In this theoretical study, we cannot address the question to what extent such a mechanism is responsible for the activation of T cell populations in vivo. However, our simulations show that the need for paracrine cytokine signals provides several checkpoints for the induction of immune responses downstream of the T cell receptor.

We identified three major control points which are likely important for the fine-tuned regulation of paracrine cytokine signals. First, cytokine receptors have high affinity and are internalized after binding of cytokine molecules. That allows for control of paracrine cytokine signals by expression of cytokine receptors (Fig 1). Second, the effective rate of cytokine secretion, i.e. the paracrine cytokine signal escaping the immunological synapse, sensitively depends on the configuration of the immunological synapse, in terms of the exact synaptic distance (Fig 2). Therefore, we propose that regulation of cytokine signals is an important function of the immunological synapse (see also Refs. [15–18]), along with regulation of the strength of T cell receptor activation [47] and the exchange of microvesicles between T cell and APC [58]. Note that using the synaptic distance is an idealization; in reality the influence of the immunological synapse on cytokine diffusion is more complex, due to its structure consisting of several layers with different types of surface proteins [16,24]. Third, in our simulations, Treg cells efficiently suppress paracrine IL-2 signals, because they express high basal levels of IL-2R, preventing the strong transient cytokine signal. In line with earlier work from us and others [4,5,29,57], this suggests that suppression of IL-2 signals is an important mechanism contributing to immune tolerance mediated by Treg cells. Of note, Treg cells most likely interfere with T cell activation in several other ways, e.g. by release of anti-inflammatory cytokines like IL-10, by forming immunological synapses with T cells, and by other mechanisms yet to be discovered [12,33]. Interestingly, a fourth system property one might expect to have a large influence on the dynamics of the system, the cell density or cell-to-cell distance, is unimportant for the results of our simulations (Fig 4E). This property results from the timescale separation between cytokine diffusion and cytokine uptake (see Fig 1E and S1 Text), and explains recent experimental data [7].

In our model simulations, paracrine cytokine signals are not only characterized by stable cytokine gradients, but also by a rapid and transient cytokine boost occurring in the first hours after stimulation. Such a transient cytokine signal has been observed by single-cell IL-2 capture assays [49,59], and recently also by ELISA in cell supernatants [7], although with conflicting time-scales: IL-2 capture assays evoked a peak in the number of IL-2 secreting cells at 1–6 hr after antigen stimulation [49,59], while Tkach et al. report a peak in the IL-2 concentration measured in vitro after ~50 hr [7]. Our simulations point to an IL-2 peak in the first 10 hr after stimulation, and thus support the earlier suggestion [49] that ELISA studies have limitations in reflecting the time-course of *in vivo* cytokine signals, although the study of Tkach et al. provides valuable quantitative insight into the dose-response characteristics of IL-2 signals. A reason might be that in culture, cells form thin layers on the bottom of the well, and therefore cytokine molecules are detected by ELISA in the supernatant after a certain delay.

The large-scale simulations resembling a cluster of highly active T cells in the center of a lymphoid organ (Fig 5) reveals a logarithmic, or fold-change response of the spatial signal range with respect to the effective secretion rate. That means, the cell population recognizes relative rather than absolute increases in the stimulus strength (here, the amount of secreted cytokine molecules per time). Fold-changes in sensory biological systems are a classical phenomenon referred to as Weber’s law, and were recently observed in various intracellular signal transduction pathways [60–63]. As a consequence of the fold-change response, sensory systems can act over a broad range of stimulus intensities, from nearly detectable to very intense stimulations. Our computer simulations suggest a similar mechanism for paracrine cytokine signals: Moderate effective secretion by a small fraction of cells allows for short-range signals inside an immunological synapse, larger effective secretion rates may evoke paracrine signals that reach bystander cells in close vicinity but not connected by a synapse, and very high secretion rates or large clusters of secreting cells may evoke an organ-wide cytokine signal or ‘cytokine storm’ [14].

Adaptive immune responses must be rapid and effective in the case of strong infection, but also carefully controlled to avoid autoimmune diseases. In our simulations, the spatial distribution of cytokine secretion and uptake within a population of immune cells had a huge impact on the cellular response, generating multiple layers of plasticity that can be exploited for appropriate regulation of immune responses.

## Materials and Methods

### Software

For the simulations of the three-dimensional in silico T cell population (Figs 3–5), a problem specific software was developed in the Heidelberg Numerical Methods Group, based on the open source C++ library deal.II [41]. The system was discretized in time by the damped Crank-Nicolson method. The intercellular area was discretized with an unstructured adaptive mesh, which describes each cell with at least 342 degrees of freedom (64 in the long range simulations in Fig 5) by a Galerkin approach using continuous finite elements (Q1). The discretized system was solved efficiently by controlling the error with adaptive space and time grids by means of the Dual Weighted Residual (DWR) method[42,44]. To allow for larger time steps, the equations were solved in a fully coupled fashion and not with the commonly applied iterative segregating approach. We linearized the nonlinear equations with Newton's method and applied Krylov-Space methods (GMRES) with a geometric multilevel preconditioner [40,43] to solve the resulting linear equations. In the simulations, the secreting Th cells and the Treg cells and the synapse on the cell surface of secreting cells were positioned randomly. We checked the influence of this cell positioning on the simulations with different randomly chosen positions and found that the variations between simulations were negligible.

Our discretized high-resolution numerical data were visualized in cooperation with the Visualization and Numerical Geometry Group from the Interdisciplinary Center of Scientific Computing (IWR) in Heidelberg. For the graphical representation of the three-dimesional scalar data, here the IL-2 distribution in space, two methods were applied, the visualization of isosurfaces using topological methods [64,65] and volume rendering [64]. With the first method specific isosurfaces are visualized by varying the transparency for different isovalues to get an impression of the 3D data set (Figs 5A, S1 and S2). To choose these specific isosurfaces with important features, topological information (Morse complex, persistent homology classes and Betti numbers) is computed. The rendering was performed by using the Visualization Toolkit VTK (http://www.vtk.org) which allows rotation in real time. The second method, volume rendering, produces the image directly from the data without an intermediate geometrical representation. A play with transparency of the whole data set makes the inner structures visible (Figs 3D and 4D). With flexible mapping of the data on colors and opacity, different structures can be visualized efficiently and a realistic representation is obtained (Figs 3C and 3D and 4C and 4D). Difficulties in the data-representation were the wide range of the values over several orders of magnitude and the porous domain (extracellular domain).

The simulations for pulsed stimulation (Fig 5D and S3 Fig) were realized by homogeneous secretion by the cell in the center of the region for a very short time (7 sec) with a q_eff_ such that a concentration corresponding to a single cytokine molecule is released. The simulation is then run until the concentration reaches zero in the whole area. The fraction of the released IL-2 concentration bound by a certain responder cell is equivalent to the probability that the ‘secreted molecule’ was bound. This probability was calculated for the successive layers of responder cells surrounding the secretory cell, in order to obtain the distribution of the traveling distance.

Analytical calculations were supported by Wolfram’s Mathematica. Matlab from Mathworks was used to generate plots and to calculate the special functions applied in Fig 2.

### Characteristic time of cytokine signaling

A classical formula derived by Berg and Purcell approximates the characteristic time τ of a ligand diffusing towards a receptor [48]:

$$\tau =\frac{1}{4\pi D\rho c}+\frac{1}{4Dd_{R}Rc}=6.9\min$$ (1)

Here, we suppose a cytokine concentration of c = 10 pM, a receptor diameter of d_R =_ 0.1nm, a receptor number of R = 100 per cell, and diffusion constant D and cell radius ρ as in Table 1. Note that in Eq 1 and in the following, cytokine concentrations (nM) are implicitly converted to molecules/μm^3^ by Avogadro’s constant N_A_, wherever necessary, as follows: nM = 10^-9^mol/l = 10^-9^N_A_ molecules/(10^15^μm^3^) = 6/10 molecules/μm^3^. Note that the time to diffuse towards a T cell (first term in Eq 1) is less than a second, but the mean time to reach a receptor at the cell surface (second term in Eq 1) is in the order of minutes due to the small number of receptors on naïve T cells.

### Homogeneous cytokine secretion and uptake

One cytokine secreting cell is either surrounded by a layer of responder cells (‘high cell-density’, see Fig 1B) or placed in a cell-free medium (‘low cell-density’). The cytokine secreting cell has R cytokine receptors, and responder cells have R_resp_ cytokine receptors, both binding cytokine molecules in their immediate vicinity with rate k_on_. We assume homogeneous cytokine secretion and uptake, so that the system has radial symmetry. As diffusion is fast (D = 10μm^2^/s, see Table 1), it reaches a steady state after about L^2^/D = 0.5 s, where L is the cell-to-cell distance in the case of high cell-density. Thus, it is sufficient to consider the diffusion equation in steady state in the extracellular domain with flux boundary condition at the cell surface:

$$\begin{array}{l} D\Delta c(r)=0,\;\;r\in [\rho ,\infty ]\\ -4\pi \rho^{2}D\frac{\partial c}{\partial r}{|}_{r=\rho }=q-k_{on}c(\rho )R \end{array}$$ (2)

c(r) is the cytokine concentration at distance r from the center of the cell, Δ is the Laplace operator in spherical coordinates, ρ is the cell radius, and q is the cytokine secretion rate. Note that cytokine concentrations are implicitly converted from unit nM to unit molecules/μm^3^, as above. The boundary condition on the outer boundary is either (low cell-density limit)

c(r→∞)=0 (3)

or (high cell-density limit)

$$-4\pi {\left(L+\rho \right)}^{2}D\frac{\partial c}{\partial r}{|}_{r=L+\rho }=k_{on}c(L+\rho )NR_{resp},$$ (4)

where N is the number of IL-2 consuming responder cells. In both cases, the problem can be solved analytically for the cytokine concentration c(r) and eventually for the uptake rates *J*_*auto*_ = *k*_*on*_ *c*(ρ)*R*, *J*_*para*_ = *q* − *J*_*auto*_ (see S1 Text).

### Cytokine diffusion in the immunological synapse

We consider stationary cytokine diffusion in a cylindrical region between a cytokine secreting Th cell and a responder cell, both potentially expressing cytokine receptors (see Fig 2A). This leads to the following boundary conditions at the cytokine secreting cell (z = 0) and the responder cell (z = l):

$$\begin{array}{l} D\Delta c(r,z)=0,\;r\in [0,a],\;z\in [0,l]\\ -\pi a^{2}D\frac{\partial c}{\partial z}{|}_{z=0}=q-k_{on}Rc{|}_{z=0}\\ -\pi a^{2}D\frac{\partial c}{\partial z}{|}_{z=l}=k_{on}R_{resp}c{|}_{z=l} \end{array}$$ (5)

The synaptic distance is l = 20 nm, and the radius of the contact area is a = 2 μm (see Table 1), corresponding to the region where localized IL-2R expression is reported [11]. At the outer boundary of the synapse, we assume c(a,z) = 0, which means that cytokine molecules which escape the cylindrical region do not return to it. The cytokine concentration, and the uptake rates J_auto_, J_escape_ and J_synapse_ resulting from this model, can be calculated analytically using Bessel functions (see S1 Text).

### In silico T cell population

We performed simulations in three spatial dimensions (see section ‘software’ above) of our earlier model [4], with some modifications: We consider polarized IL-2 secretion and autocrine uptake at the immunological synapse, by assuming an effective secretion rate at one grid point at the surface of IL-2 secreting cells. Moreover, due to recent experimental observations [5,35], we discard the previously assumed positive feedback from IL-2 uptake to IL-2 secretion, and we set the IL-2 secretion rate to 10 molecules/s and the fraction of IL-2 secreting cells to about 25% (see Table 1). In brief, the model [4] considers interactions of three kinds of cells: Secretory Th cells, responder Th cells and Treg cells. All three cell types express IL-2R molecules on the cell surface. Responder Th cells and Treg cells express IL-2R homogeneously at the cell surface, Treg cells at higher levels than responder Th cells. IL-2 signaling leads to the expression of the α subunit of the IL-2 receptor that is required for high-affinity IL-2 binding in both responder Th cells and Treg cells. Hence both cell types enhance their rate of IL-2R expression (*v*) upon IL-2 uptake, which we model, following Busse et al. [4], by a Hill equation with a moderate Hill coefficient of 3:

$$v(t)=v_{0}+v_{1}\frac{C{\left(t\right)}^{3}}{K^{3}+C{\left(t\right)}^{3}}$$ (6)

Here, *v*_0_ and *v*_1_ are the basal and the IL-2 induced rates of IL-2R expression, *K* is the half-saturation constant, and *C*(t) is the number of IL-2/IL-2R complexes, which is a dynamic variable of the model (Table 1). For details and the full model see S1 Text.

## Supporting Information

S1 Text Supplementary Methods. (PDF)

S1 Fig Relates to Fig 3. Alternative visualization of simulations shown in Fig 3D–3E, in terms of isosurfaces of the IL-2 concentration (see Section Materials and Methods).
(PDF)

S2 Fig Relates to Fig 4. (A) Alternative visualization of simulations shown in Fig 4C and 4D, in terms of isosurfaces of the IL-2 concentration (see Section Materials and Methods). (B-C) Time course of receptor number and IL-2 concentration at the cell surface, see Fig 3E and 3F.
(PDF)

S3 Fig Relates to Fig 5. Comparison of cytokine traveling distances (Fig 5D) with earlier analytical results by Coppey et. al. [51]. According to their theory, the probability of a ligand binding in this limited domain amounts to 64%, therefore we normalized the results of the 3D-simulations to this amount for the comparison to the theoretical probability distribution. Our simulations comply best with Coppey et al. in the center of the distribution, i.e. in the vicinity of the maximum. The larger somewhat number of ligand trapping points at the outer boundary of the simulated domain can be explained by the limited number of simulated cells. The smaller number close to the secretion source stems from the difference between an analytical model of spatially homogenized receptors and our discrete model with receptors only on the surface of individual cells.
(PDF)
