# Supplementary material for: Three-Dimensional Gradients of Cytokine Signaling between T Cells
Source: PLoS Comput Biol. 2015 Apr 29;11(4):e1004206. doi: 10.1371/journal.pcbi.1004206 (PMC4414419; doi:10.1371/journal.pcbi.1004206)
Supplement: S1 Text — (PDF) [file pcbi.1004206.s001.pdf]

Supplementary Text S1 for Article: Three-dimensional gradients of cytokine signaling between T cells

Steady-state Models of Cytokine Secretion and Uptake with Spherical Symmetry

The general solution to the stationary diffusion equation with radial symmatry is $c(r) = A/r + B$ where A and B are determined by the boundary conditions (Equations 2-4 in the main text). In low cell-density scenario, i.e. $c(r \to \infty) = 0$, the solution is

$$c(r) = \frac{q\rho}{r(k_{\text{on}}R + 4\pi D\rho)}. \tag{1}$$

In the high cell-density scenario with prescribed uptake rate at a certain cell distance L (Equation 4 in main text), the solution is

$$c(r) = \frac{q\rho}{k_{\text{on}}r} \frac{4\pi Dr(L+\rho) + k_{\text{on}}(L-r+\rho)NR_{\text{resp}}}{k_{\text{on}}LRNR_{\text{resp}} + 4\pi D\rho(L+\rho)(R+NR_{\text{resp}})}, \tag{2}$$

where ρ the cell radius, and other parameters are as in Table 1. This yields $c(r) = q/(k_{\text{on}}R + k_{\text{on}}NR_{\text{resp}})$ for fast diffusion ($D \to \infty$), as mentioned in Section Results (see Figure 1). In the case of very high receptor levels ($R \gg R_{\text{resp}}$), the solution simplifies to the constant value $c(r) = q/(k_{\text{on}}R)$ for both low and high cell-density limits, which is the steady-state value obtained without considering cytokine diffusion.

Simple expressions can also be obtained for the autocrine and paracrine uptake rates (see Figure 1). In the low cell-density limit,

$$J_{\text{auto}} = q\frac{k_{\text{on}}R}{k_{\text{on}}R + 4\pi D\rho} \tag{3}$$

$$J_{\text{para}} = q\frac{4\pi D\rho}{k_{\text{on}}R + 4\pi D\rho}. \tag{4}$$

In this case, the flux ratio is determined by the ratio of the uptake capacity $k_{\text{on}}R$ and the term $4\pi D\rho$ which describes dilution in the surrounding medium. For high cell-density,

$$J_{\text{auto}} = q\frac{R(k_{\text{on}}LNR_{\text{resp}} + 4\pi D\rho(L+\rho)}{k_{\text{on}}LRNR_{\text{resp}} + 4\pi D\rho(L+\rho)(R+NR_{\text{resp}})} \xrightarrow{D\to\infty} q\frac{R}{R+NR_{\text{resp}}} \tag{5}$$

$$J_{\text{para}} = q\frac{4\pi D\rho NR_{\text{resp}}(L+\rho)}{4\pi D\rho(R+NR_{\text{resp}})(L+\rho) + k_{\text{on}}LRNR_{\text{resp}}} \xrightarrow{D\to\infty} q\frac{NR_{\text{resp}}}{R+NR_{\text{resp}}}. \tag{6}$$

Therefore, the fluxes are nearly independent of the cell-to-cell distance L also in the high cell-density scenario, and depend largely on the ratio of receptor numbers R and R_{resp} (Figure 1). For high receptor levels R, the autocrine signal tends to q in both limiting cases, i.e. almost all cytokine molecules are recaptured before escaping the vicinity of the cytokine secreting cell.

Steady-state Model of Cytokine Signaling in the Immunological Synapse

Exact solution

The diffusion problem in cylindrical coordingates with absorbing boundary condition $I(r,a) = 0$ has the general solution [1]

$$I(r,z) = \sum_{n=1}^{\infty} J_0(\alpha_n r)[A_n \cosh(\alpha_n z) + B_n \sinh(\alpha_n z)], \tag{7}$$

where $J_i(x)$ is the i-th order Bessel function, and the α_n, $n = 1 \ldots \infty$, are the positive roots of $J_0(\alpha a)$. The coefficients A_n and B_n are computed from the boundary conditions at the top and bottom of the synapse (see main text Equations 5),

$$-\pi a^2 D \frac{\partial c}{\partial z}|_{z=0} = q - k_{\text{on}} R c|_{z=0} \tag{8}$$

$$-\pi a^2 D \frac{\partial c}{\partial z}|_{z=l} = k_{\text{on}} R_{\text{resp}} c|_{z=l}. \tag{9}$$

This is a standard problem, which can be solved by substitution of Equation 7, multiplication with $rJ_0(\alpha_m r)$, and performing $\int_{r=0}^{a} dr$ in each boundary condition. We can then use the properties of Bessel functions,

$$\int_0^a rJ_0(\alpha_n r)J_0(\alpha_m r)dr = \begin{cases} 0, & m \neq n \\ \frac{a^2}{2} J_1(\alpha_m a), & m = n \end{cases} \tag{10}$$

$$\int_0^a rJ_0(\alpha_m r)dr = \frac{a}{\alpha_m} J_1(\alpha_m a). \tag{11}$$

The coefficients read

$$A_m = 2q \frac{\alpha_n \cosh(\alpha_n l) + p \sinh(\alpha_n l)}{a\alpha_n J_1(\alpha_n r)[\alpha_n k_{\text{on}}(R + R_{\text{resp}}) \cosh(\alpha_n l) + (a^2 \alpha_n^2 D\pi + k_{\text{on}} R p) \sinh(\alpha_n l)]} \tag{12}$$

$$B_m = -2q \frac{p \cosh(\alpha_n l) + \alpha_n \sinh(\alpha_n l)}{a\alpha_n J_1(\alpha_n r)[\alpha_n k_{\text{on}}(R + R_{\text{resp}}) \cosh(\alpha_n l) + (a^2 \alpha_n^2 D\pi + k_{\text{on}} R p) \sinh(\alpha_n l)]}, \tag{13}$$

where $p = (k_{\text{on}} R_{\text{resp}})/(\pi D a^2)$.

Calculation of cytokine fluxes

Based on the exact solution described above, we can calculate the cytokine fluxes discussed in the main text, which are

$$J_{\text{auto}} = D \int_{z=0} \frac{\partial c}{\partial z} dA - q = k_{\text{on}} \frac{R}{\pi a^2} \int_{z=0} c(r,z) dA \tag{14}$$

$$J_{\text{synapse}} = -D \int_{z=l} \frac{\partial c}{\partial z} dA = k_{\text{on}} \frac{R_{\text{resp}}}{\pi a^2} \int_{z=l} c(r,z) dA \tag{15}$$

$$J_{\text{escape}} = -D \int_{r=a} \frac{\partial c}{\partial r} dA = q - J_{\text{auto}} - J_{\text{para}}. \tag{16}$$

The surface integrals in the equations for J_{auto} and J_{para} can be solved analytically in polar coordinates, by substitution of Equations 7 and 11. This leads to the following expressions:

$$\begin{aligned} J_{\text{auto}} &= 4qk_{\text{on}}R \sum_{n=1}^{\infty} \frac{\alpha_n \cosh(\alpha_n l) + p \sinh(\alpha_n l)}{K_n \alpha_n} \\ J_{\text{synapse}} &= 4qk_{\text{on}}R_{\text{resp}} \sum_{n=1}^{\infty} \frac{1}{K_n} \\ J_{\text{escape}} &= 4\pi D a^2 q \sum_{n=1}^{\infty} \frac{p \cosh(\alpha_n l) + \alpha_n \sinh(\alpha_n l) - p}{K_n}, \end{aligned} \tag{17}$$

with

$$\begin{aligned} K_n &= a^2\alpha_n[\alpha_n k_{\text{on}}(R + R_{\text{resp}}) \cosh(\alpha_n l) + a^2\alpha_n^2 \pi D \sinh(\alpha_n l) + k_{\text{on}} R p \sinh(\alpha_n l)] \\ p &= \frac{k_{\text{on}} R_{\text{resp}}}{\pi a^2 D}. \end{aligned} \tag{18}$$

Note that

$$J_{\text{auto}} + J_{\text{synapse}} + J_{\text{escape}} = \sum_{n=1}^{\infty} \frac{4q}{a^2\alpha_n^2} = q, \tag{19}$$

where the last equation sign holds because $\sum_n 1/(\alpha_n a)^2 = 1/4$.

Model of Cytokine Signaling in a T Cell Population in Three Spatial Dimensions

We reconsider a model previously analyzed in two dimensions [2] with some modifications, as outlined in Section Materials and Methods. As this model contains

detailed receptor dynamics which are specific for IL-2, we now denote the cytokine concentration by I for [IL-2]. This leads to the following reaction-diffusion problem with two different types of boundary conditions for IL-2 secreting and non-secreting cells, respectively:

$$\frac{\partial I}{\partial t} = D\Delta I - k_d I \tag{20}$$

$$-D\frac{\partial I}{\partial n} = -q_{\text{eff}}(s) \tag{21}$$

$$-D\frac{\partial I}{\partial n} = k_{\text{on}} R I(s,t) - k_{\text{off}} C. \tag{22}$$

n is the normal vector pointing into the cell, and $q_{\text{eff}}(s)$ denotes polarised IL-2 secretion at one randomly chosen point at the cell surface. The size of the simulated region is defined by the number of cells and the cell-to-cell distance. In the standard setup (Figures 3-4), we simulated a cubic region with periodic boundary conditions.

Homogeneous receptor expression implies that receptor dynamics depend on the IL-2 concentration in the surrounding medium averaged over the cell surface, $\langle I \rangle_{\text{surf}}$. At the cell surface, IL-2 is bound to IL-2R molecules (R), forming IL-2/IL-2R complexes (C). Receptor dynamics involve receptor expression (rate v); binding (unbinding) of IL-2 to (from) the receptor (k_{on}, k_{off}); internalisation of bound and unbound receptor (k_{iC} and k_{iR}); recycling and degradation of bound receptors (k_{rec}, k_{deg}). Thus, receptor dynamics are governed by a set of nonlinear differential equations,

$$\begin{aligned}
\frac{dR}{dt} &= v - (k_{\text{on}}\langle I \rangle_{\text{surf}} + k_{\text{iR}})R + k_{\text{off}}C + k_{\text{rec}}E \\
\frac{dC}{dt} &= k_{\text{on}} R \langle I \rangle_{\text{surf}} - (k_{\text{off}} + k_{\text{iC}})C \\
\frac{dE}{dt} &= k_{\text{iC}} C - (k_{\text{rec}} + k_{\text{deg}})E.
\end{aligned} \tag{23}$$

Receptor expression is enhanced by a positive feedback from bound receptors, which is modeled by a Hill function with moderate nonlinearity:

$$v = v_0 + v_1 \frac{C^3}{K^3 + C^3}. \tag{24}$$

In Eq. 24, v_0 is the basic IL-2R expression rate and v_1 is the expression rate dependent on IL-2 uptake, which drives T cell activation. Both parameters have different values for Th and Treg cells (Table 1). The chosen parameter values lead to steady state receptor numbers of active or inactive Th or Treg cells similar to those used in Figs. 1-2 with the analytic models. The parameter values not given in Table 1 of the main paper are taken from Ref. [2].

References

[1] Carslaw HS, Jaeger JC (1986) Conduction of Heat in solids. Oxford: Oxford University Press.

[2] Busse D, de la Rosa M, Hobiger K, Thurley K, Flossdorf M, et al. (2010) Competing feedback loops shape IL-2 signaling between helper and regulatory T lymphocytes in cellular microenvironments. Proc Natl Acad Sci U S A 107: 3058-63.
