# Supplementary figures and images for: Three-Dimensional Gradients of Cytokine Signaling between T Cells
Source: PLoS Comput Biol. 2015 Apr 29;11(4):e1004206. doi: 10.1371/journal.pcbi.1004206 (PMC4414419; doi:10.1371/journal.pcbi.1004206)

1h

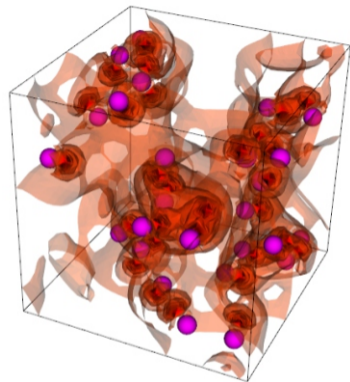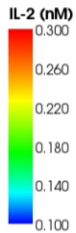

9h

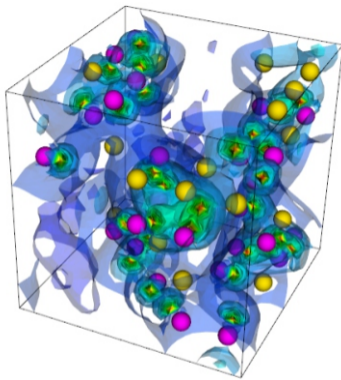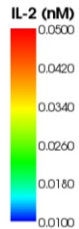

30h

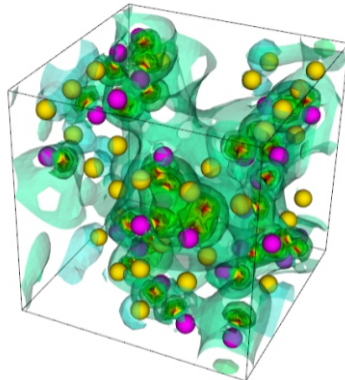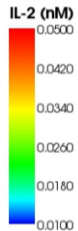

Supplement: S1 Fig — Alternative visualization of simulations shown in Fig 3D–3E, in terms of isosurfaces of the IL-2 concentration (see Section Materials and Methods). (PDF) [file pcbi.1004206.s002.pdf]

A

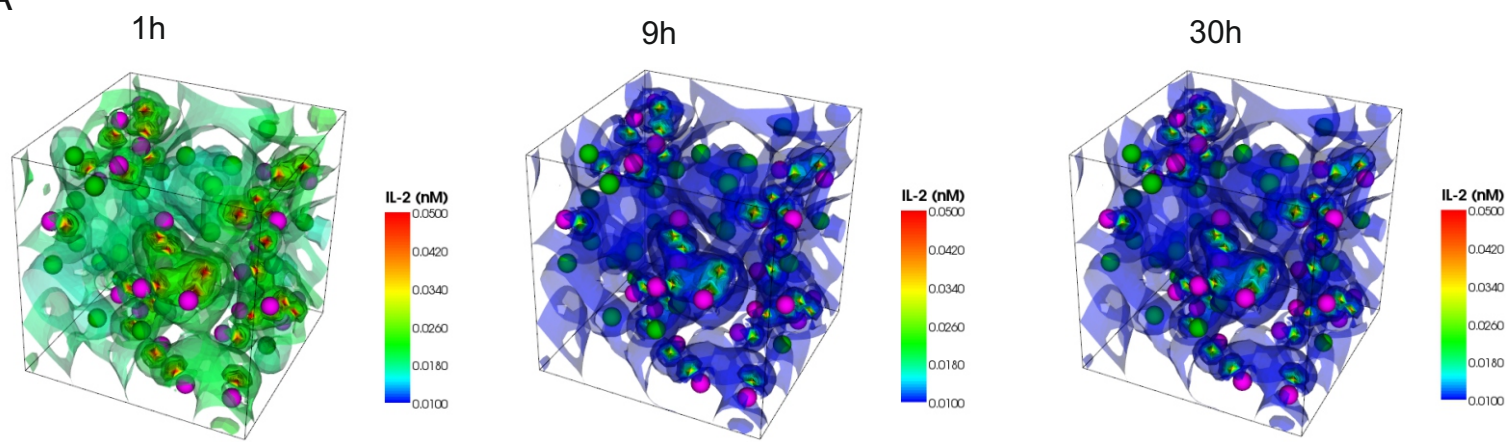

B

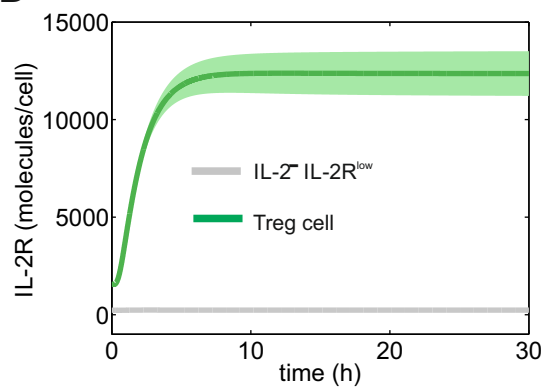

C

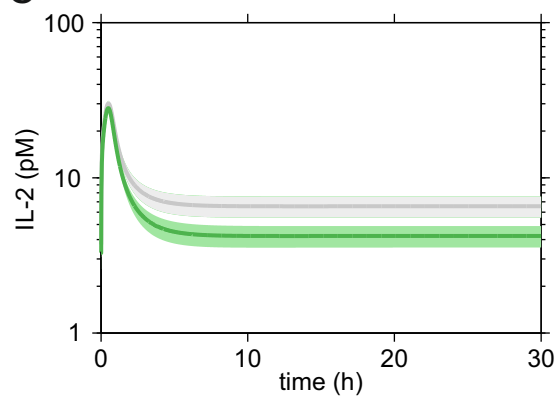

Thurley et al. Figure S2

Supplement: S2 Fig — (A) Alternative visualization of simulations shown in Fig 4C and 4D, in terms of isosurfaces of the IL-2 concentration (see Section Materials and Methods). (B-C) Time course of receptor number and IL-2 concentration at the cell surface, see Fig 3E and 3F. (PDF) [file pcbi.1004206.s003.pdf]

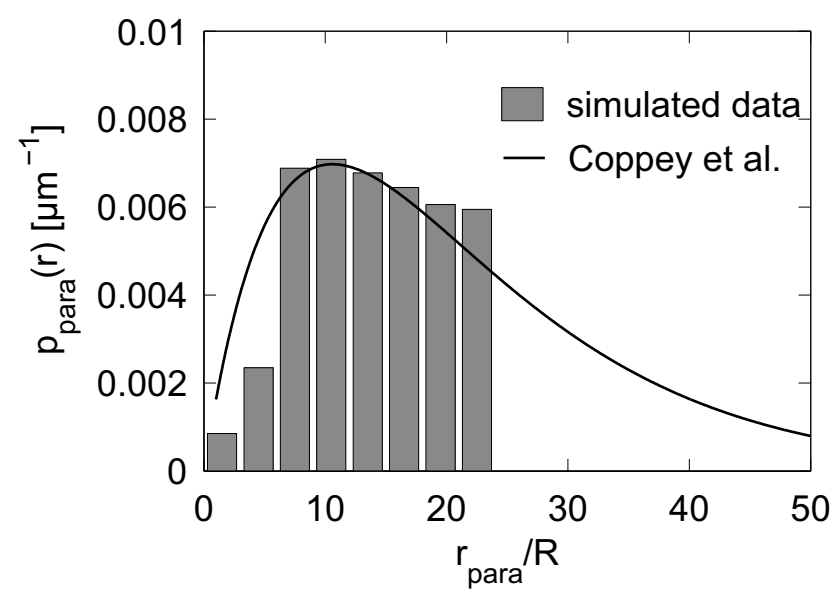

Thurley et al. Figure S3

Supplement: S3 Fig — Comparison of cytokine traveling distances (Fig 5D) with earlier analytical results by Coppey et. al. [51]. According to their theory, the probability of a ligand binding in this limited domain amounts to 64%, therefore we normalized the results of the 3D-simulations to this amount for the comparison to the theoretical probability distribution. Our simulations comply best with Coppey et al. in the center of the distribution, i.e. in the vicinity of the maximum. The larger somewhat number of ligand trapping points at the outer boundary of the simulated domain can be explained by the limited number of simulated cells. The smaller number close to the secretion source stems from the difference between an analytical model of spatially homogenized receptors and our discrete model with receptors only on the surface of individual cells. (PDF) [file pcbi.1004206.s004.pdf]
